# Catching the virus – a peer-to-peer game that encourages active participation in microbiology lectures

**DOI:** 10.1099/acmi.0.000302

**Published:** 2022-01-13

**Authors:** Paul Dean

**Affiliations:** ^1^​ School of Health and Life Sciences, Teesside University, Middlesbrough, UK

**Keywords:** COVID-19, virus, Hoberman, game, microbiology

## Abstract

An important part of learning within lectures and classrooms is active participation, but this is sometimes difficult in larger lecture rooms. Questioning students is also not very fruitful in larger rooms for many reasons and invariably results in a wall of silence. Playing active-learning games changes the student–teacher dynamic and energizes the lecture room, making the lecture more memorable and worthwhile for the students. In our microbiological lectures, particularly lectures on virology and immunology, students play the ‘catch-the-virus’ game. As all students are in the game together, there is a competitive edge, and students forget about the anxiety of the the lecture theatre. Importantly, because of the nature of the game, the entire lecture room is involved, including students in the back rows. Interestingly, the recent coronavirus disease 2019 (COVID-19) pandemic, and its impact on student lives, makes the catch-a-virus game even more poignant.

## Introduction

Low-level student participation and passive learning in lectures can have a negative effect on learning in the lecture room [1–5]. Active teaching approaches also increase attendance and engagement in science, technology, engineering and mathematics (STEM) subjects [5]. Therefore, finding new ways for students to raise their concentration and increase participation is challenging for the lecturer, particularly in larger lecture rooms, as active participation is often associated with increased anxiety, particularly in the sciences [6]. By using games to engage students, teachers can encourage higher levels of participation; and by introducing competition, anxiety in the room may diminish naturally. The ‘catch-the-virus’ game was developed specifically for viral lectures and has been adopted in several microbiological classes at the University of Teesside. Students must catch an expanding virus and answer a range microbiology-based questions. The game is controlled by the students in class as each student selects the next student to answer the question. Such empowerment adds an interesting dynamic to the lecture. The game is enjoyable for staff and students and greatly increases class participation.

## Methods

The Hoberman sphere is an expanding sphere that looks like a viral capsid. It has the appearance of a spherical virus and expands from 9 inches to over 30 inches when in the air. While any ball could substitute, the Hoberman sphere is light and safe to catch and looks like a virus. As it expands through the air, it looks far more impressive than a regular ball. The sphere can be purchased for around £30 in the UK.

The room is divided into two and students are initially given a question and told to think about the answer. The lecturer then throws the virus into the room, and the catcher must answer the first question. A correct answer allows them to throw the virus to another student, who will then need to answer the next question. A wrong answer will be passed to the opposing team, who can confer and provide the answer. Each team has 10 questions, and scores are written on the whiteboard. Prizes or rewards for the winning team make the game more competitive. The game lasts about 30 min. Variations of the game can include students posing questions to their peers – although the students will probably need prior warning.

The success of the game prompted feedback and data acquisition during the activities. Data on student participation was collected in-class by recording student involvement in answering the questions posed, compared with conventional question and answer (e.g. raising hand) sessions. Students that took part in the lectures were all level 3 and 4 undergraduates at Teesside University. In addition to student involvement, the student location in the lecture room was determined by recording the different number of benches on which students sat. This gave a measure of student involvement across the lecture room. Data were collected over four semesters.

## Results

**Fig. 1.**
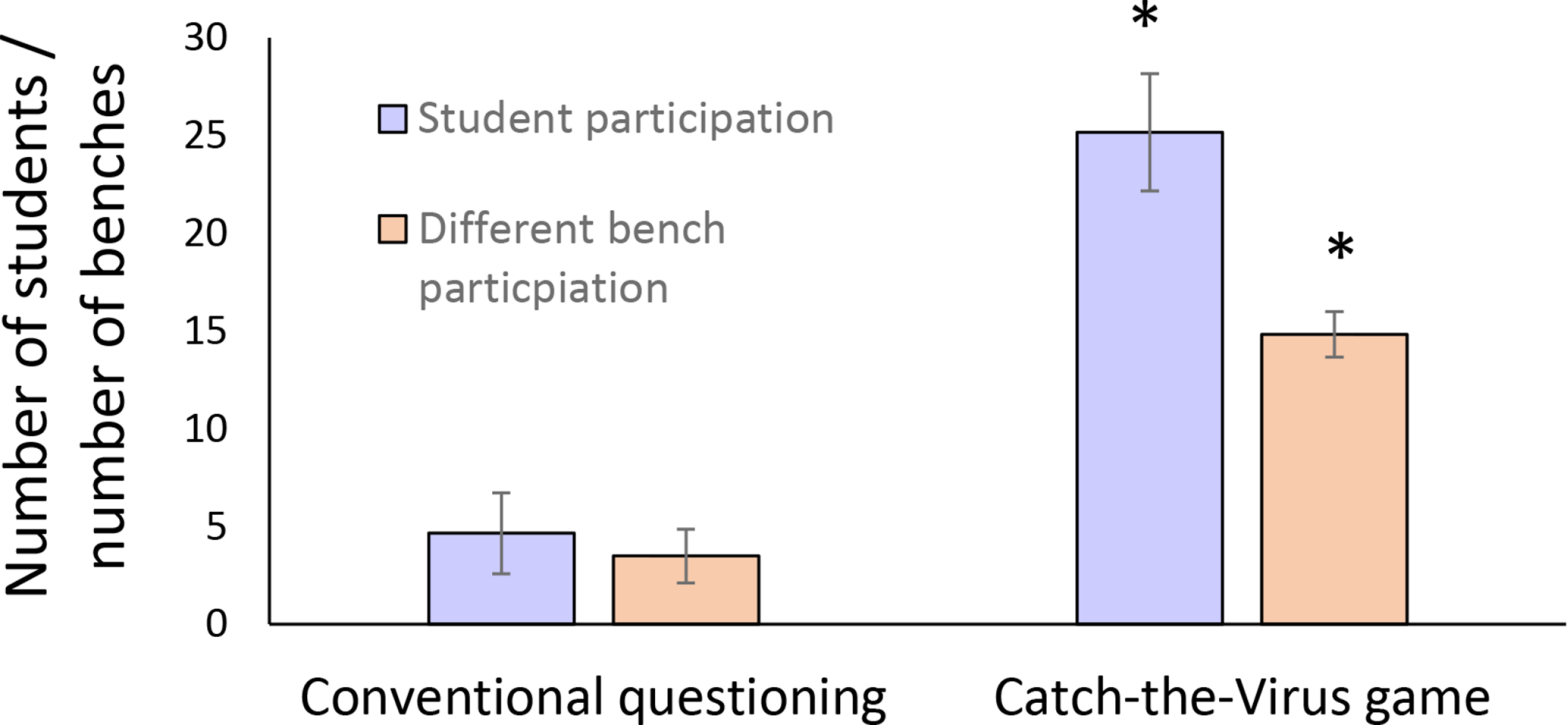
Changes in student participation during the catch-the-virus game in immunology and microbiology lectures. Student participation was recorded as the number of different students that answered questions posed in the lecture. Bench participation refers to the number of different benches involved in answering the questions (front row=bench 1 and so on). Students were level 3 and 4 life science undergraduates at Teesside University. Data show the mean±SD from six separate lectures. Data shows the mean±SD. *, significant difference (*P*<0.01) between both sets of data.

As shown in Fig. 1, the catch-the-virus game increased student participation significantly, over fivefold when compared to conventional question-and-answer sessions. Student involvement across the room, including the back benches, was also measured and was low during traditional question-and-answer sessions, but increased significantly, as shown by the increase in the number of different benches involved (Fig. 1).

The overall perception of the students in the lecture is that they soon forgot anxiety and wanted to actively take part. Whether or not they got the questions right was secondary, as they are having fun playing the game. The room dynamics quickly changed upon playing the game, involving the whole room, and it provided an excellent and productive mechanism for breaking up the lecture.

## Conclusion

Encouraging participation in the lecture room is one of the most challenging tasks for a lecturer. Combining playful games like catch-the-virus with more serious lecture content, stirs the competitive spirit in students, and many soon forget the confines of the lecture room. Further, for microbiological and virology lectures, throwing a viral-like sphere in the air ensures students are constantly reminded about the viral capsid and topology, and analogies can be made concerning how quickly a virus can spread in the population. With the recent impact of coronavirus disease 2019 (COVID-19), and the massive interest and uptake in virology classes, the catch-a-virus game has more significance and can play an important part in these lectures.
